# Distribution and Uniqueness in the Pattern of Lip Prints

**DOI:** 10.7759/cureus.53692

**Published:** 2024-02-06

**Authors:** Muralidhar Reddy Sangam, Praveen K, Raju R Bokan, Vinay G, Amandeep Kaur, Roonmoni Deka

**Affiliations:** 1 Anatomy, All India Institute of Medical Sciences, Guwahati, Guwahati, IND

**Keywords:** identification, uniqueness, lip score, sex differences, lip print pattern

## Abstract

Introduction

Lip prints are the characteristic pattern of wrinkles and grooves on the labial mucosa. Lip prints can be classified into various patterns and can be used for personal identification as they are unique and do not change during the life of a person. Cheiloscopy is a forensic investigation technique that deals with the identification of humans based on lip traces.

Objectives

This study aimed to investigate the distribution of lip print patterns, to assess gender differences, and to calculate the lip score using a weighted value scoring system.

Material and methods

A cross-sectional study was carried out in the Department of Anatomy, All India Institute of Medical Sciences (AIIMS), Guwahati, India, from May to October 2023, after getting approval from the Institutional Ethics Committee (IEC). A total of 200 individuals (100 males and 100 females) were included in the study. Each lip print was divided into four quadrants. In each quadrant, up to 14 grooves were marked from the midline, and the pattern of each groove was observed. Each pattern was given an Arabic numeral score. Weighted values were given for the grooves in descending order from 15 to 1 with reference to their position from the midline of the lip print. The product of the Arabic numeral score of the groove and the weighted value of the groove is the lip line score. The sum of the lip line scores was calculated.

Results

The most common pattern observed in the present study is type II, with 3,816/12,000 (31.8%), followed by type I’ with 3,146/12,000 (26.21%), type I with 1,865/12000 (15.54%), type III with 1,491/12,000 (12.42%), type IV with 1,133/12,000 (9.44%), and type V with 549/12,000 (4.5%). The mean total lip score is 1,467.68 (1,486.41 in males and 1448.96 in females).

Conclusion

Lip prints are unique and useful for personal identification, as the lip score in various quadrants and the total lip score are different for different individuals.

## Introduction

The identity of a person is a prerequisite for various personal, social, and legal issues [1]. Dermatoglyphics, anthropometry, odontology, and blood groups are the proven methods of identification of a person. A number of forensic studies revealed that prints produced by the lips when they are impressed on a surface at the crime scene form a source of personal identification [2].

Lip prints are the characteristic pattern of wrinkles and grooves on the labial mucosa. Cheiloscopy is a forensic investigation technique that deals with the identification of humans based on lip traces [3]. These grooves, which appear in the sixth week of intrauterine life, are unique and do not change during the life of a person [4]. The grooves on the red part of the lip were first described by R. Fischer [5]. Edmond Locard [6] and Le Moyne Synder [7] recommended the use of lip prints for the identification of a person. Santos was the first person to classify the pattern of lip prints into four types: straight line, curved line, angled line, and sine-shaped line [8]. Suzuki and Tsuchihashi classified the pattern of lip prints into six types: type I (a clear-cut groove running vertically across the lip), type I′ (a partial-length groove of type I), type II (a branched groove), type III (an intersected groove), type IV (a reticular pattern), and type V (other patterns) [9]. The four-quadrant method was suggested by Tsuchihashi to study lip prints. In this method, a lip print was divided into four quadrants in a clockwise direction: upper right (quadrant I), upper left (quadrant II), lower left (quadrant III), and lower right (quadrant IV) [10]. To establish its uniqueness, Prabhu et al. [11] calculated the lip score using a weighted value scoring system.

Although many studies described the distribution and sex differences in the pattern of lip prints, very few studies [11] have described the uniqueness of the pattern of lip prints. Lip prints are unique as they vary in number, length, thickness, and branching. The present study aimed at studying the distribution in the pattern of lip prints and establishing the uniqueness of lip prints using a weighted value scoring system.

Objectives

First, this study aimed to investigate the distribution of lip print patterns and assess gender differences in lip print patterns. Second, it aimed to calculate the total lip score using a weighted value scoring system to establish the uniqueness of the lip prints.

## Materials and methods

This cross-sectional study was carried out in the Department of Anatomy, All India Institute of Medical Sciences (AIIMS), Guwahati, India, from May to October 2023. The study was approved by the Institutional Ethics Committee of AIIMS, Guwahati (Ref. No. AIIMSG/IEC/M1/F1/2023 dated February 20, 2023).

**Figure 1:**
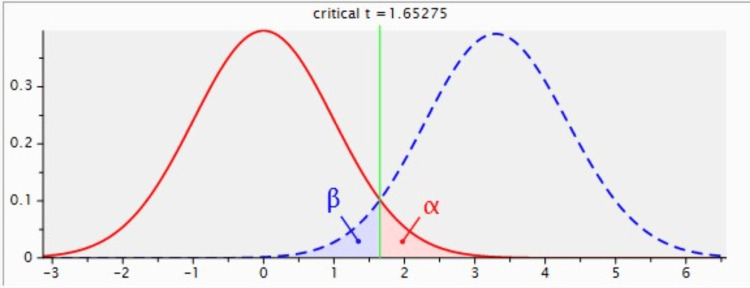
Sample size calculation using the G*Power software

The study comprised 200 healthy individuals (100 males and 100 females) in the age group of 17 to 25 years and belonging to different regions of India. The sample size was calculated using the G*Power computer software (written by Franz Faul, Universitat Kiel, Germany) [12]. A total sample size of 196 was calculated to detect a small effect (d = 0.20). The power of the test was fixed at 90% and alpha at 0.05 (Fig. 1).

Informed consent was obtained from all the subjects. All participants who gave voluntary consent were included in the study. Participants with known hypersensitivity to lipsticks, participants with surgical scars, and those without consent owing to religious and personal reasons were excluded from the study.

Method of data collection

**Figure 2:**
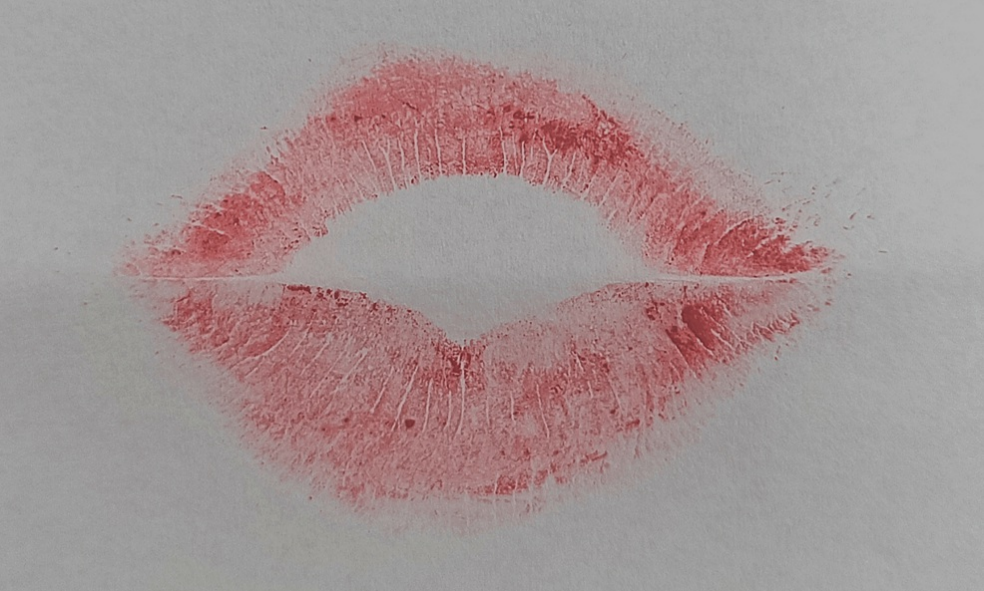
Lip print

A lipstick (Revlon, Revlon, Inc., USA), cellophane tape, white chart paper, magnifying glass, and tissue paper were used. The upper and lower lips of the individual were cleaned with wet cotton gauze, and the lipstick was applied uniformly to the lips. A white piece of paper was used for the impression of the lip prints. A hinged portion of the folded paper was placed between the lips, and they were instructed to press their lips by applying pressure evenly. The paper was then unfolded (Fig. 2).

Then, a glued portion of a cellophane strip was pasted on the imprinted white paper for better preservation. A lipstick remover was given to the participants to clean their lips. The lip prints collected were labeled with the identification number, age, and gender. The pattern of each lip print was studied using a magnifying glass. 

Analysis of the lip print

**Figure 3:**
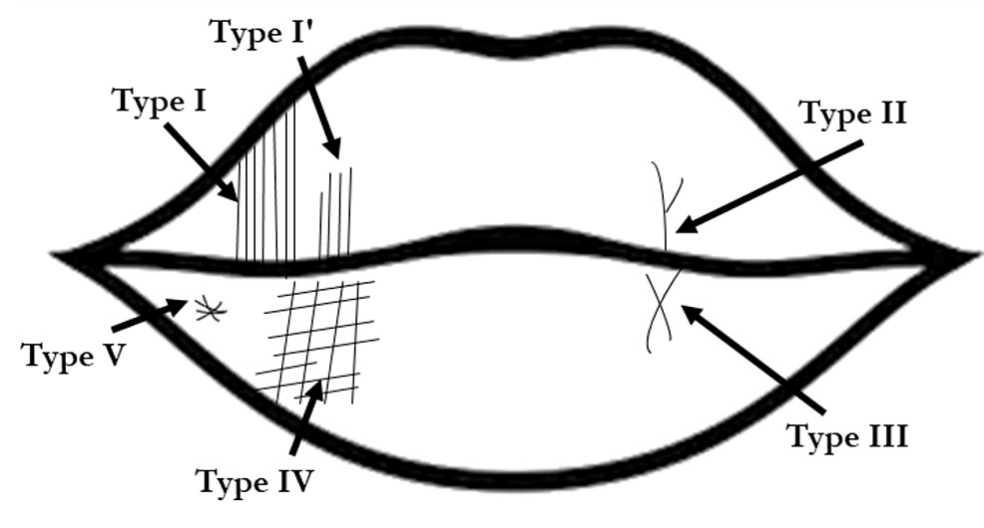
Pattern of lip prints The figure shows the Suzuki and Tsuchihashi classification of lip prints [9]. Type I: a clear-cut groove running vertically across the lip; type I′: a partial-length groove of type I; type II: a branched groove; type III: an intersected groove; type IV: a reticular pattern; and type V: other patterns.

In this study, the classification of patterns of lines on the lips (Fig. 3) proposed by Suzuki and Tsuchihashi [9] was used.

Each lip print was divided into four quadrants: upper right (quadrant I), upper left (quadrant II), lower left (quadrant III), and lower right (quadrant IV). In each quadrant, up to 15 grooves were marked from the midline, and the pattern of each groove was observed. Each pattern in the Suzuki and Tsuchihashi classification was given an Arabic numeral score (Table 1).

**Table 1:** Arabic numeral scoring of each pattern of lip prints in the Suzuki and Tsuchihashi classification. Citation: Prabhu et al.  [11]

Lip print pattern	Arabic numeral scoring
Type I - clear-cut groove running vertically across the lip	1
Type I’ - partial-length groove of type I	2
Type II - branched groove	3
Type III - intersected groove	4
Type IV - reticular pattern	5
Type V - other patterns	6

To calculate the lip score, weighted values were given for the grooves in descending order from 15 to 1 with reference to their position from the midline of the lip print, as described by Prabhu et al. [11]. More weightage was given to the grooves close to the midline of the lip print, as that portion of the lip is mostly imprinted. The product of the Arabic numeral score of the groove and the weighted value of the groove is the lip line score. The sum of lip line scores in each quadrant was calculated. The total lip score of an individual was calculated by summing up the lip line scores in all four quadrants.

Statistical analysis

Data analysis was done with the help of IBM SPSS Statistics for Windows, version 21 (released 2012; IBM Corp., Armonk, New York, United States). The data are presented in frequency distribution and percentage.

## Results

The present study includes 200 individuals (100 males and 100 females) from various parts of North India. The quadrant-wise distribution of lip prints in the entire study population is shown in Table 2. In the present study, the type II pattern was more common. The distribution of lip prints in the upper lip was type II > type I’ > type I > type III > type IV > type V. In the lower lip, the distribution was type I’ > type II > type I > type III > type IV > type V.

**Table 2:** Distribution of lip print patterns (n = 200).

Pattern	Q1 (15X200)	Q2 (15X200)	Q1+Q2 (30X200)	Q3 (15X200)	Q4 (15X200)	Q3+Q4 (30X200)	Q1+Q2+Q3+Q4 (60X200)
Type I	469 (15.63%)	419 (13.96%)	888 (14.8%)	519 (17.3%)	458 (15.26%)	977 (16.28%)	1865 (15.54%)
Type I’	616 (20.53%)	601 (20.03%)	1217 (20.28%)	975 (32.5%)	954 (31.8%)	1929 (32.15%)	3146 (26.21%)
Type II	1002 (33.4%)	1065 (35.5%)	2067 (34.5%)	863 (28.76%)	886 (29.53%)	1749 (29.15%)	3816 (31.8%)
Type III	382 (12.73%)	395 (13.16%)	777 (12.95%)	358 (11.93%)	356 (11.86%)	714 (11.9%)	1491 (12.42%)
Type IV	352 (17.6%)	358 (11.93%)	710 (11.83%)	192 (6.4%)	231 (7.7%)	423 (7.05%)	1133 (9.44%)
Type V	179 (5.96%)	162 (5.4%)	341 (5.68%)	93 (3.1%)	115 (3.83%)	208 (3.46%)	549 (4.5%)

The distribution of lip prints among males is shown in Table 3. In males, the general distribution of lip prints was type II> type I’ > type I > type III > type IV > type V. The distribution of lip prints in the upper lip was type II > type I’ > type I > type IV > type III > type V, and in the lower lip, the distribution was type I’ > type II > type I > type III > type IV > type V.

**Table 3:** Distribution of lip print patterns in males (n = 100).

Pattern	Q1 (15X100)	Q2 (15X100)	Q1+Q2 (30X100)	Q3 (15X100)	Q4 (15X100)	Q3+Q4 (30X100)	Q1+Q2+Q3+Q4 (60X100)
Type I	246 (16.4%)	223 (14.8%)	469 (15.63%)	265 (17.66%)	235 (15.66%)	500 (16.66%)	969 (16.15%)
Type I’	264 (17.6%)	284 (18.93%)	548 (18.26%)	522 (34.8%)	454 (30.26%)	976 (32.53%)	1524 (25.4%)
Type II	495 (33%)	513 (34.2%)	1008 (33.6%)	408 (27.2%)	448 (29.86%)	856 (28.53%)	1864 (31.06%)
Type III	199 (13.2%)	208 (13.86%)	407 (13.56%)	176 (11.73%)	186 (12.4%)	362 (12.06%)	769 (12.81%)
Type IV	209 (13.93%)	205 (13.66%)	414 (13.8%)	88 (5.8%)	120 (8%)	208 (6.93%)	622 (10.36%)
Type V	87 (5.8%)	67 (4.46%)	154 (5.13%)	41 (2.73%)	57 (3.8%)	98 (3.26%)	252 (4.2%)

The distribution of lip prints among females is shown in Table 4. In females, the general distribution of lip prints was type II> type I’ > type I > type III > type IV > type V. The distribution of lip prints in the upper lip was type II > type I’ > type I > type IV > type III > type V, and in the lower lip, the distribution was type I’ > type II > type I > type III > type IV > type V.

**Table 4:** Distribution of lip print patterns in females (n = 100).

Pattern	Q1 (15X100)	Q2 (15X100)	Q1+Q2 (30X100)	Q3 (15X100)	Q4 (15X100)	Q3+Q4 (30X100)	Q1+Q2+Q3+Q4 (60X100)
Type I	223 (14.86%)	196 (13.06%)	419 (13.96%)	254 (16.93%)	223 (14.86%)	477 (15.9%)	896 (14.93%)
Type I’	352 (23.46%)	317 (21.13%)	669 (22.3%)	453 (30.2%)	500 (33.33%)	953 (31.76%)	1622 (27.03%)
Type II	507 (33.8%)	552 (36.8%)	1059 (35.3%)	455 (30.33%)	438 (29.2%)	893 (29.76%)	1952 (32.53%)
Type III	183 (12.2%)	187 (12.4%)	370 (12.33%)	182 (12.13%)	170 (11.33%)	352 (11.73%)	722 (12.03%)
Type IV	143 (9.53%)	153 (10.2%)	296 (9.8%)	104 (6.93%)	111 (7.4%)	215 (7.16%)	511 (8.51%)
Type V	92 (6.13%)	95 (6.33%)	187 (6.23%)	52 (3.46%)	58 (3.86%)	110 (3.66%)	297 (4.95%)

The data were analyzed using the Chi-square test. A statistically significant difference was observed in the distribution of lip prints between the males and females in quadrants 1 and 2 (Table 5).

**Table 5:** Statistical analysis of the distribution of lip print patterns among the males and females using the Chi-square test. *P <0.05 statistically significant, X2 – chi-square

	Q1	Q2	Q1+Q2	Q3	Q4	Q3+Q4	Q1+Q2+Q3+Q4
X^2^	27.027	18.48	40.67	10.41	3.72	2.54	23.98
Df	5	5	5	5	5	5	5
Probability	0.0005*	0.002*	1.09	0.064	0.589	0.769	0.0002*

No two lip prints were identical in the present study. In none of the cases, there is an exact matching of the lip score in all the quadrants. The comparative distribution of the mean lip score between the males and females is shown in Table 6.

**Table 6:** Comparison of the mean lip score in the males and females. Data are expressed as mean ± standard deviation.

Quadrant	Males (n = 100)	Females (n = 100)	Total (n = 200)
Q1	396.54±109.70	367.97±122.16	382.25±194.58
Q2	392.11±96.37	371.35±125.58	381.73±184.02
Q1+Q2	788.65±201.19	739.32±247.18	763.98±375.41
Q3	358.07±112.78	356.11±113.34	354.82±219.52
Q4	339.69±113.63	353.53±111.65	348.88±210.89
Q3+Q4	697.76±218.80	709.64±224.15	703.7±426.28
Q1+Q2+Q3+Q4	1486.41±300.01	1448.96±322.12	1467.68±552.16

## Discussion

The study of lip prints is called cheiloscopy. The present study was conducted to study the distribution of lip print patterns quadrant-wise, to assess the differences in the lip print pattern among males and females, and to establish the uniqueness of lip prints by calculating the total lip score.

In the present study, the type II pattern of lip prints was the most common type (31.8%), followed by type I’ (26.21%), type I (15.54%), type III (12.42%), type IV (9.44%), and type V (4.5%). A comparative description of the distribution of lip prints in various studies is given in Table 7.

**Table 7:** Comparative study of the distribution of lip prints in various studies.

S. No.	Study	Population	Sample size	Type I	Type I’	Type II	Type III	Type IV	Type V
1	Tsuchihashi [2]	Japanese	64	26.56%	-	21.87%	32.81%	12.5%	6.2%
				III > I > II > IV > V					
2	Augustine et al., 2008 [13]	Maharastra, Indian	600	11.10%	2.54%	18.92%	48.2%	17.44%	1.58%
				III > II > IV > I > I’ > V					
3	Abhishek et al., 2015 [14]	Nepalese	150	48.61%	12.5%	31.25%	2.77%	2.77%	2.08%
				I > II > I’ > III = IV > V					
4	Prabhjot et al., 2016 [15]	Haryana ethnic, Indian	-	6%	0.9%	35.4%	16.6%	37%	4.06%
				IV > II > III > I > V > I’					
5	Venkat Rao et al., 2016 [16]	Telangana, Indian	300	35.33%	20%	10.66%	11.66%	16%	6.33%
				I > I’ > IV > III > II > V					
6	Remya et al., 2016 [17]	Kerala, India	200	10.5%	23.5%	22%	10.5%	26%	7.5%
				IV > I’ > II > I = III > V					
7	Kapoor et al., 2017 [18]	Marathi, Indian	200	30.63%	1.88%	16.5%	25.38%	8.63%	17%
				I > III > V > II > IV > I’					
8	Archana et al., 2017 [19]	Telangana, Indian	100	55%	-	32%	1%	7%	5%
				I > II > IV > V > III					
9	Omer HAM 2019 [20]	Yemen	84	15.67%	8.1%	43.8%	16.1%	8.5%	7.73%
				II > III > I > IV > I’ > V					
10	Loganadan et al., 2019 [21]	Deutero Malay, Indonesia	-	1.39%	30.28%	8.33%	14.44%	28.89%	16.67%
				I’ > IV > V > III > II > I					
11	Timsinha et al., 2019 [22]	Nepalese	100	7.25%	11.25%	75.25%	2.75%	3.5%	0
				II > I’ > I > IV > III > V					
12	Baral et al., 2020 [23]	Nepalese	150	25.3%	-	17.3%	29.3%	23.3%	4.7%
				III > I > IV > II > V					
13	Okeke et al., 2020 [24]	Nigerian	300	26.7%	-	27.3%	19.7%	15%	11.3%
				II > I > III > IV > V					
14	Chawla et al., 2023 [25]	North India	200	17.25%	18%	20.25%	22.25%	17.5%	4.75%
				III > II > I’ > IV > I > V					
15	Present study	Indian	200	16.5%	26.21%	31.8%	12.42%	9.44%	4.5%
				II > I’ > I > III > IV > V					

In the present study, the distribution of lip prints in both males and females is type II > type I’ > type I > type III > type IV > type V. Analysis of lip prints using the chi-square test revealed that the difference in the pattern of lip prints between males and females is statistically significant with a P value <0.05 in quadrant 1, quadrant 2, and combined. There is no significant difference in quadrants 3 and 4 between males and females. Kumar et al. [26], Koneru et al. [27], and Dwivedi et al. [28] observed statistically significant differences in the lip print pattern between males and females. Sandhu et al. [29] and Prabhu et al. [11] did not observe any statistically significant differences in the lip print pattern between males and females.

In the upper lip quadrants (Q1 and Q2), the distribution of lip prints is type II > type I’ > type I > type III > type IV > type V. However, in the lower lip, the distribution is type I’ > type II > type I > type III > type IV > type V. In the lower lip, type I’ is the most common type of pattern in both males and females.

To determine the uniqueness of lip prints, a total lip score was calculated. In the present study, no two lip prints have the same total lip score. There are a few cases where the lip scores in a particular quadrant are the same but the lip scores in other quadrants are different. In none of the cases, the lip score in all the quadrants was exactly matching. The patterns of lip prints are unique to an individual and can be used as an identification tool.

Limitations

As lip prints are produced by the mobile part of the lip, different prints may be recorded from the same person depending upon the pressure and method of recording. The quality of a lip print is affected by the amount of lipstick applied. Smudging of lip prints is a major limitation.

## Conclusions

Lip prints are characteristic of an individual, and like fingerprints, they have the potential for individual identification. The conclusions of the present study are as follows: Lip prints have a quadrant-wise predilection; the most common pattern of lip print is type II, followed by type I’, I, III, IV, and V in that order. Moreover, there is a significant difference in the distribution of lip prints between males and females in quadrant 1, quadrant 2, and combined. Finally, the total lip score is different in different individuals, which describes the uniqueness of the lip prints.
